# Prognostic relevance of the triglyceride–glucose index in patients after thrombectomy for acute anterior circulation occlusion

**DOI:** 10.3389/fneur.2025.1625856

**Published:** 2025-08-14

**Authors:** Zhongxiu Wang, Siyuan Wang, Chao Li, Kangjia Song, Xiaoyu Wu, Yu Jiang, Mingchen Zhang, Shouchun Wang

**Affiliations:** 1 Center for Rehabilitation Medicine, Department of Neurology, Zhejiang Provincial People’s Hospital (Affiliated People’s Hospital, Hangzhou Medical College), Hangzhou, Zhejiang, China; 2 Department of Neurology and Neuroscience, The First Hospital of Jilin University, Changchun, Jilin, China

**Keywords:** intravascular treatment, mechanical thrombectomy, ischemic stroke (acute), large vessel occlusion, triglyceride-glucose (TyG) index

## Abstract

**Background:**

We aimed to investigate the relationship between the triglyceride–glucose (TyG) index and the 3-month prognosis after mechanical thrombectomy (MT) in patients with acute large-vessel occlusion in the anterior circulation.

**Methods:**

We performed a retrospective analysis of data collected from 320 patients who underwent MT at our institution between May 2022 and January 2024. The main outcome measure was the modified Rankin Scale (mRS) score, with a score of ≤2 at 90 days post-treatment indicating a good prognosis. Secondary outcomes were the 90-day mRS score distribution, 24-h postoperative National Institutes of Health Stroke Scale (NIHSS) score, and NIHSS score at discharge. Safety outcomes were 90-day mortality, intracranial hemorrhage (symptomatic and asymptomatic), and surgical complications.

**Results:**

One hundred and eight patients (33.8%) achieved functional independence at 90 days. After adjusting for covariates, patients who fell within the second group of the TyG index exhibited a lower probability of functional independence than those in the first group (adjusted odds ratio [aOR] = 0.450; 95% confidence interval [CI], 0.257–0.789; *p* = 0.005). Additionally, an increase of one unit in the TyG index was significantly correlated with a 33% reduction in the likelihood of achieving functional independence at 90 days postoperatively (aOR = 0.669; 95% CI: 0.450–0.996; *p* = 0.048).

**Conclusion:**

This study demonstrates that the TyG level was significantly related to achieving functional independence within 90 days among patients who have undergone MT for acute anterior circulation infarction. Based on traditional neurological function (e.g., NIHSS) assessments, the TyG index may be used as an independent prognosis predictor after thrombectomy in patients with acute ischemic stroke.

## Introduction

Ischemic stroke accounts for approximately 70% of all stroke cases and represents a primary factor contributing to long-term disability and fatality rates. Large vessel occlusion often leads to acute ischemic stroke (AIS), which tends to be severe and has a poor prognosis (1, 2). For patients with AIS stemming from large vessel occlusion, early endovascular treatment improves functional outcomes and reduces 90-day disability. Nevertheless, over 50% of patients undergoing mechanical thrombectomy (MT) have a poor clinical prognosis (3, 4). Elevated random or fasting glucose levels at admission correlate with poor prognosis in patients with AIS undergoing MT or thrombolysis (5, 6). Moreover, increased levels of triglycerides (TGs) constitute a prominent risk factor for cardiovascular and cerebrovascular diseases (CVDs) (7).

Insulin resistance (IR), characterized by reduced insulin sensitivity, serves as an independent predictor of ischemic stroke and contributes to its onset, progression, and prognosis by promoting thrombosis and atherosclerosis (8, 9). This condition is closely associated with various CVD risk factors, including atherosclerosis, hypertension, type 2 diabetes mellitus, coronary heart disease, and atrial fibrillation (10–13). The high insulin-positive glucose clamp test is the gold standard for evaluating IR (14). However, because of its complexity and high cost, the triglyceride–glucose (TyG) index, which combines blood glucose (BG) and TG levels, has become a valuable alternative for assessing IR (15). A significant association has been demonstrated between TyG levels and the Homeostatic Model Assessment of Insulin Resistance (HOMA-IR) and high insulin-normal glucose clamp test (16), with the TyG index potentially outperforming the HOMA-IR in predicting certain diseases (17). Additionally, TyG levels are strongly linked to an elevated risk of detrimental cardiovascular occurrences in both individuals with high-risk factors and the general population (18). In patients with ST-segment elevation myocardial infarction (STEMI) treated via percutaneous coronary intervention, elevated TyG levels were also linked to a poorer clinical prognosis (19). However, its prognostic value for patients with severe anterior circulation stroke undergoing MT remains uncertain. This study adds to the existing literature by showing that elevated TyG levels are associated with poorer 90-day functional outcomes following mechanical thrombectomy in patients with acute large-vessel occlusion, a relationship not previously established. This novel finding suggests that TyG may serve as a prognostic marker for patients undergoing MT, beyond its established role in predicting cardiovascular events.

## Methods

### Study population

We retrospectively analyzed a prospective cohort of patients with AIS who underwent MT at Jilin University First Hospital between January 2022 and January 2024. The First Hospital of Jilin University’s Ethics Committee approved the study protocol. The need for informed consent was waived due to the anonymity of the clinical data analysis. The research procedures adhered to the ethical standards outlined in the Declaration of Helsinki. The inclusion criteria were acute large-vessel occlusive stroke in the anterior circulation, confirmed via imaging techniques such as magnetic resonance imaging (MRI), computed tomography (CT), and intraoperative digital subtraction angiography. We excluded patients with (1) spontaneous intracranial hemorrhage (e.g., hypertensive cerebral hemorrhage, ruptured aneurysm hemorrhage) confirmed by preoperative CT/MRI of the head while those with postoperative hemorrhagic transformation (e.g., symptomatic intracranial hemorrhage [sICH]) were retained; (2) acute posterior circulation ischemic stroke; (3) a pre-stroke modified Rankin Scale score ≥2, as these patients were considered to have pre-existing disability, which could confound the results of this study; and (4) missing essential clinical information, such as follow-up information or critical baseline information.

### Data collection

Demographic and baseline clinical characteristics of the patients were collected by reviewing electronic medical records. These including sex, age, cholesterol levels (total cholesterol [TC], TG, high- [HDL-C] and low-density lipoprotein cholesterol [LDL-C]), systolic and diastolic blood pressure (SBP and DBP), homocysteine, urea nitrogen, serum creatinine, BG, glycosylated hemoglobin, and various medical conditions including hypertension, diabetes, atrial fibrillation, transient ischemic attack, wake up stroke, intracerebral hemorrhage, and coronary artery disease.

Additional information included the smoking status, drinking status, blood pressure upon admission, baseline NIHSS score, Alberta Stroke Project Early Computed Tomography Score (ASPECTS) upon admission, site of vascular occlusion, stroke etiology, use of intravenous thrombolysis or tirofiban, anesthesia modality, American Society of Interventional and Therapeutic Neuroradiology/Society of Interventional Radiology (ASITN/SIR) score <2, and presence of tandem lesions. Laboratory tests, including BG, glycosylated hemoglobin, and blood pressure measurements, were performed within 24 h of admission. Stroke etiology was categorized based on the Trial of Heparin-like Drug Therapy for Acute Ischemic Stroke Staging. The duration between stroke inception and revascularization was recorded. sICH was defined as intracranial hemorrhage confirmed by cranial CT/MRI within 24 h of surgery, accompanied by an increase of ≥4 points in NIHSS score or new neurological deficits. If the time of onset was unknown, it was considered to be the last known normal time. All imaging was assessed independently by two blinded neuroradiologists. A third investigator assessed and confirmed the results in cases of inconsistent assessment.

### Laboratory investigations

TG and BG were collected from fasting venous blood at the time of admission (prior to thrombectomy), before the patients received any intravenous fluids containing glucose. The BG concentration was measured using the enzymatic hexokinase method. TG, TC, LDL-C, and HDL-C, were assayed enzymatically with a fully automated biochemistry analyzer. The TyG index was calculated using the following formula: ln [fasting TGs (mg/dL) × BG (mg/dL)/2] (20). Because TG, BG, and TyG are highly correlated, the model prioritizes the inclusion of the composite indicator TyG to simplify the analysis.

### Study outcomes

The key efficacy indicator was the 90-day functional independence of patients post-thrombectomy, reflected by an mRS score of 0–2. Secondary efficacy indicators encompassed the 90-day mRS, 24-h postoperative NIHSS, and discharge NIHSS scores. Safety metrics included surgery-related complications (arterial perforation, distal embolization, and arterial entrapment), any intracranial hemorrhage (ICH) within 48 h, sICH within 24 h, and 90-day mortality. The mRS is scored on a 7-point categorical scale assessing neurological disability from asymptomatic (0) to death (6). Functional improvement is defined as a one-grade mRS score reduction (21). The 90-day mRS score was obtained through telephone or outpatient follow-up, and the mRS scores were assessed by a blinded neurologist trained in the neurology department.

### Statistical analyses

To assess quantitative variables, the Shapiro–Wilk test was initially employed to verify normality, revealing that none of the variables in this study were normally distributed. Median and interquartile ranges (interquartile ranges [IQRs]) were used for non-normal distributions, whereas the Kruskal–Wallis H or Wilcoxon Mann–Whitney U tests were employed for comparative analyses.

**Figure 1:**
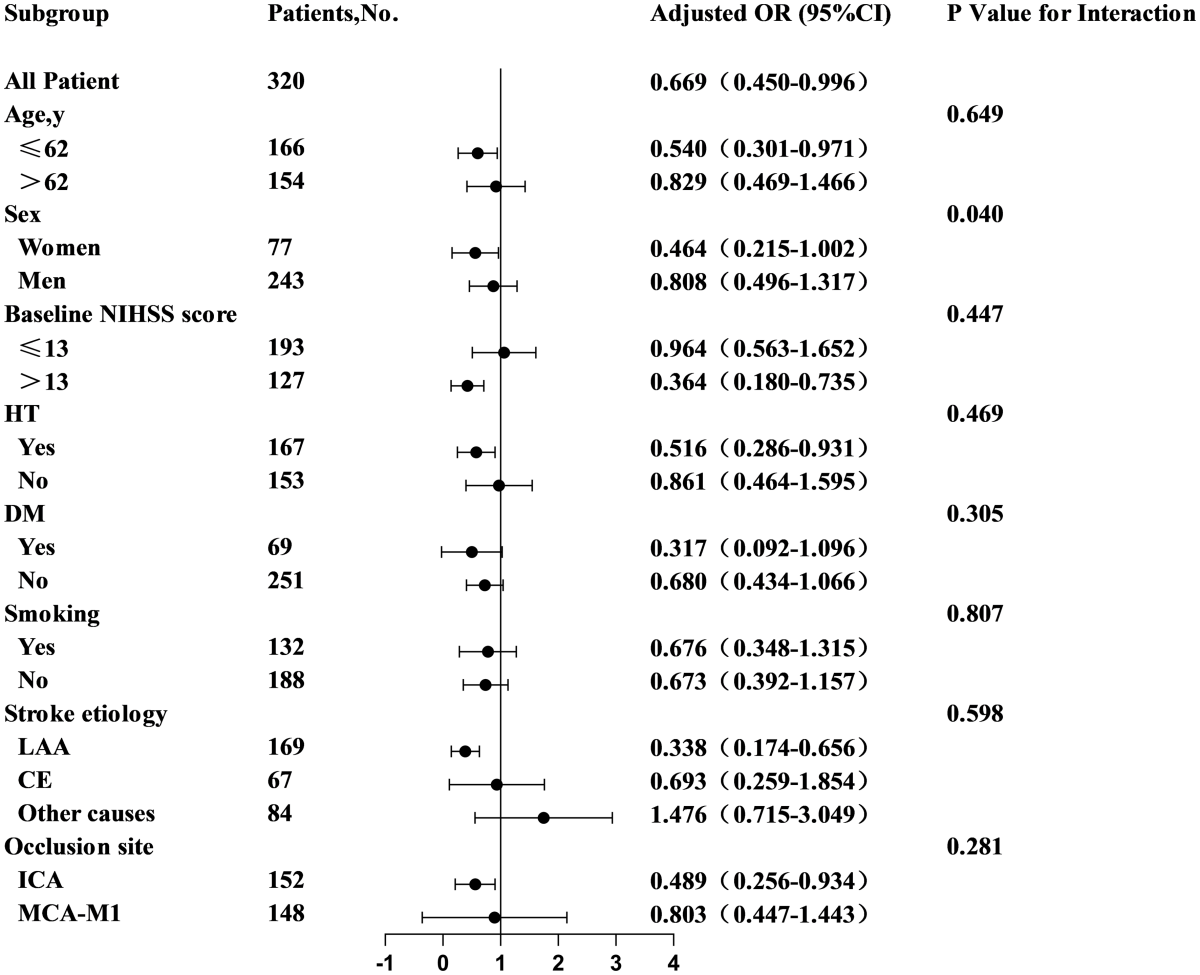
Subgroup analyses of clinical outcomes. Forest plots show that the difference in correlation between TyG and favorable outcome in subgroups of patients with different characteristics at 90 days. Adjusted variables including age, sex, SBP, baseline NIHSS score, baseline ASPECTS score, CHD, diabetes, stroke etiology, occlusion site and recanalization ratio.

Qualitative variables are reported as percentages which were subject to one-way comparisons using either Fisher’s exact or chi-square tests. Binary logistic regression analysis was used to evaluate the effect of varying TyG levels on primary, secondary, and safety outcomes. Clinically pertinent factors and significant univariate covariates (*p* < 0.05) were integrated into the logistic regression models. We adjusted for covariates such as age, sex, SBP, diabetes, coronary artery disease, occlusion site, stroke etiology, baseline NIHSS scores, recanalization rates, and ASPECTS scores. Associations between TyG groups and functional independence were assessed using binary logistic regression. To analyze the associations between TyG and mRS scores, an ordered logistic regression model was used, whereas linear regression was used to evaluate the correlation between TyG and NIHSS scores post-surgery and at discharge. Sensitivity analyses via subgroup analyses were used to validate the consistency in outcomes between the TyG levels and functional independence in patients with diverse baseline characteristics. Figure 1 presents all odds ratios (ORs) with 95% confidence intervals (CIs).

Statistical evaluations were performed using SPSS Statistics v. 25 (IBM Corp.), with significance levels determined bilaterally and statistical significance set at *p* < 0.05 for all conducted tests. Multiple imputation (*n* = 5) was employed to handle missing data. The proportion of missing data for key variables ranged from 0 to 10%. No permissible range for missing values was pre-specified during the study design phase, but the decision to use imputation was based on the observed patterns of missingness in the dataset.

## Results

### Baseline demographic and clinical characteristics

A cohort of 356 patients with acute anterior circulation lesions underwent MT from January 2022 to January 2024. The 90-day functional prognosis was obtained by outpatient or telephone follow-up with a mean follow-up time of 90 ± 7 days. Thirty-six patients with missing important clinical data were excluded; consequently, 320 patients (median age, 62 [IQR, 54–70] years) who underwent emergency anterior circulation MT were included, of which 243 were men (75.9%). The median ASPECTS score was 8 (IQR, 7–10), and the median baseline NIHSS score was 13 (IQR, 10–16). The median TyG levels were 9.01 (IQR, 8.58–9.48). The TyG index was categorized into two levels based on the median value: 160 patients in the first ([Q1], 7.67–9.01) group and 160 patients in the second ([Q2], 9.02–11.71) group. Functional independence was achieved by 108 patients, and 39 patients died.

The baseline characteristics of patients grouped by median TyG are presented in Table 1 Significant differences (*p* < 0.05) were observed between the two groups in terms of age, SBP, DBP, TC, TG, LDL-C, glucose, HbA1C, hypertension, diabetes, baseline NIHSS score, and ASITN/SIR <2 scores. Differences in the remaining indices were not significant. The TyG index was positively associated with TC, LDL-C, TG, SBP, DBP, glucose, HbA1C, hypertension, diabetes, and baseline NIHSS scores and negatively correlated with age and ASITN/SIR <2 scores. Additional comprehensive patient characteristics are outlined in Table 1.

**Table 1:** Baseline characteristics of the study population by median of TyG index.

Variables	Overall (320)	Q1 (7.67–9.01) 160	Q2 (9.02–11.71) 160	*p*
Age, years	62 (54–70)	65 (55–72)	60 (52–69)	0.004
Male, *n* (%)	243/320 (75.9)	121/160 (75.6)	122/160 (76.3)	0.896
SBP, mmHg	148 (134–165)	145 (129–160)	152 (135–168)	0.014
DBP, mmHg	88 (78–97)	86 (76–95)	90 (80–100)	0.003
TC, mmol/L	4.52 (3.75–5.32)	4.21 (3.60–4.86)	4.96 (4.05–5.65)	<0.001
TG, mmol/L	1.37 (0.99–2.00)	1.01 (0.81–1.18)	1.98 (1.58–2.66)	<0.001
HDL, mmol/L	1.05 (0.88–1.24)	1.06 (0.90–1.28)	1.02 (0.87–1.20)	0.062
LDL, mmol/L	2.81 (2.30–3.39)	2.58 (2.23–3.13)	3.11 (2.53–3.66)	<0.001
Homocysteine, umol/L	12.71 (10.22–17.88)	13.14 (10.74–78.85)	12.03 (10.00–16.57)	0.1
Urea, mmol/L	5.54 (4.48–6.94)	5.36 (4.42–7.04)	5.76 (4.55–6.83)	0.619
Scr, umol/L	65.35 (54.6–75.88)	65.40 (56.50–75.58)	65.10 (52.80–76.55)	0.708
Glucose, mmol/L	7.10 (6.17–8.91)	6.51 (5.81–7.23)	8.23 (6.91–11.28)	<0.001
HbA1C, mmol/L	5.90 (5.60–6.88)	5.70 (5.50–6.00)	6.40 (5.70–7.95)	<0.001
Baseline NIHSS score	13 (10–16)	13 (11–16)	12 (10–15)	0.009
Baseline ASPECTS score	8 (7–10)	9 (7–10)	8 (6–10)	0.241
Medical history, *n* (%)				
Hypertension	167/320 (52.2)	80/160 (50.0)	87/160 (54.4)	0.433
Diabetes	69/320 (21.6)	17/160 (10.6)	52/160 (32.5)	<0.001
Smoking	132/320 (41.3)	63/160 (39.4)	69/160 (43.1)	0.496
Drinking	99/320 (30.9)	44/160 (27.5)	55/160 (34.4)	0.183
Atrial fibrillation	41/320 (12.8)	23/160 (14.4)	18/160 (11.3)	0.403
TIA	84/320 (26.3)	46/160 (28.7)	38/160 (23.8)	0.309
ICH	3/320 (0.9)	1/160 (0.6)	2/160 (1.3)	0.562
Wake up stroke	88/320 (27.5)	44/160 (27.5)	44/160 (27.5)	1
CHD	38/320 (11.9)	20/160 (12.5)	18/160 (11.3)	0.730
Stroke etiology, *n* (%)				0.077
LAA	169/320 (52.8)	83/160 (51.9)	86/160 (53.8)	
CE	67/320 (20.9)	41/160 (25.6)	26/160 (16.3)	
Other causes	84/320 (26.3)	36/160 (22.5)	48/160 (30.0)	
Occlusion site, *n* (%)				0.194
ICA	152/320 (47.5)	67/160 (41.9)	85/160 (53.1)	
MCA-M1	148/320 (46.3)	83/160 (51.9)	65/160 (40.6)	
MCA-M2	17/320 (5.3)	8/160 (5.0)	9/160 (5.6)	
Other	3/320 (0.9)	2/160 (1.3)	1/160 (0.6)	
Thrombolysis, *n* (%)	50/320 (15.6)	22/160 (13.8)	28/160 (17.5)	0.368
Tirofiban in operation, *n* (%)	186/320 (58.1)	86/160 (53.8)	100/160 (62.5)	0.113
ASITN/SIR <2, *n* (%)	158/320 (49.4)	88/160 (55.0)	70/160 (43.8)	0.044
Anesthesia, *n* (%)				0.535
General anesthetic	94/320 (29.4)	44/160 (27.5)	50/160 (31.3)	
Sedation	66/320 (20.6)	31/160 (19.4)	35/160 (21.9)	
Local anesthetic	160/320 (50.0)	85/160 (53.1)	75/160 (46.9)	
Tandem lesion, *n* (%)	72/320 (22.5)	31/160 (19.4)	41/160 (25.6)	0.181
Puncture to recanalization, min	55 (37–88)	54 (35–84)	60 (38–97)	0.081
Recanalization, *n* (%)	281/320 (87.8)	146/160 (91.3)	135/160 (84.4)	0.06

SBP, systolic blood pressure; DBP, diastolic blood pressure; TC, total cholesterol; TG, triglyceride; LDL, low-density lipoprotein; HDL, high-density lipoprotein; Scr, serum creatinine; HbA1C, glycosylated hemoglobin; CHD, coronary heart disease; TIA, transient ischemic attack; ICH, intracerebral hemorrhage; LAA, large artery atherosclerosis, CE, cardio-embolism; ICA, internal carotid artery; MCA, middle cerebral artery; NIHSS, National Institutes of Health Stroke Scale; ASPECTS, Alberta Stroke Program Early CT Score; ASTIN/SIR, ASITN/SIR grade indicates the American Society of Interventional and Therapeutic Neuroradiology/Society of Interventional Radiology Collateral Score.

### Primary outcome

Table 2 shows the association between TyG levels and clinical outcomes across different groups. The percentage of patients achieving an mRS score of 0–2 at 90 days at different TyG levels reflects 90-day functional independence. The unadjusted model revealed that higher TyG indices were correlated with a reduced likelihood of achieving functional independence at 90 days post-event (Table 2). A TyG index in the 9.02–11.71 range was correlated with a decreased likelihood of achieving functional independence (adjusted OR [aOR] = 0.450; 95% CI, 0.257–0.789; *p* = 0.005) when the TyG index was analyzed as a categorical variable (Q1: 7.67–9.01 and Q2: 9.02–11.71) and adjusted for age, sex, SBP, baseline NIHSS score, baseline ASPECTS score, coronary artery disease, diabetes, stroke etiology, occlusion site, and recanalization rate.

**Table 2:** Efficacy outcomes and safety outcomes.

Variable	Q1 (7.67–9.01, *n* = 160)	Q2 (9.02–11.71, *n* = 160)	*p*	a(c)OR/β-coefficient (95% CI)	*p*
Primary outcome					
mRS 0–2 at 90 days, *n* (%)	66 (41.3)	42 (26.3)	0.005	0.450 (0.257–0.789)	0.005
Secondary outcomes					
mRS at 90 days, median (IQR)	3 (2–4)	3 (2–4)	0.057	1.65 (1.06–2.59)	0.027
NIHSS at 24 h, median (IQR)	10 (5–14)	10 (6–14)	0.257	2.13 (0.55–3.72)	0.009
NIHSS at discharge, median (IQR)	7 (2–12)	8 (4–13)	0.101	2.04 (0.11–3.96)	0.038
Safety outcomes					
Any ICH, *n* (%)	34 (21.3)	42 (26.3)	0.294	1.795 (0.966–3.338)	0.064
sICH, *n* (%)	10 (6.3)	12 (7.5)	0.659	1.566 (0.579–4.236)	0.377
Mortality, *n* (%)	21 (13.1)	18 (11.3)	0.609	0.719 (0.308–1.678)	0.446
Surgery-related complications, *n* (%)	28 (17.5)	38 (23.8)	0.168	1.536 (0.820–2.876)	0.180

Outcome	Covariates adjusted for
Primary outcome (mRS 0–2 at 90 days)	Age, sex, SBP, baseline NIHSS score, baseline ASPECTS score, CHD, diabetes, stroke etiology, occlusion site and recanalization ratio.
Secondary outcome 1 (mRS at 90 days)	Age, sex, SBP, baseline NIHSS score, baseline ASPECTS score, CHD, diabetes, stroke etiology, occlusion site and recanalization ratio.
Secondary outcome 2 (NIHSS at 24 h)	Age, sex, SBP, baseline NIHSS score, baseline ASPECTS score, CHD, diabetes, stroke etiology, occlusion site and recanalization ratio.
Secondary outcome 3 (NIHSS at discharge)	Age, sex, SBP, baseline NIHSS score, baseline ASPECTS score, CHD, diabetes, stroke etiology, occlusion site and recanalization ratio.

Adjusted for age, sex, SBP, baseline NIHSS score, baseline ASPECTS score, CHD, diabetes, stroke etiology, occlusion site and recanalization ratio. Surgery-related complications including arterial perforation, distal embolization, and arterial entrapment. mRS, modified Rankin Scale; NIHSS, National Institutes of Health Stroke Scale; SICH, symptomatic intracranial hemorrhage.

### Secondary endpoints

**Figure 2:**
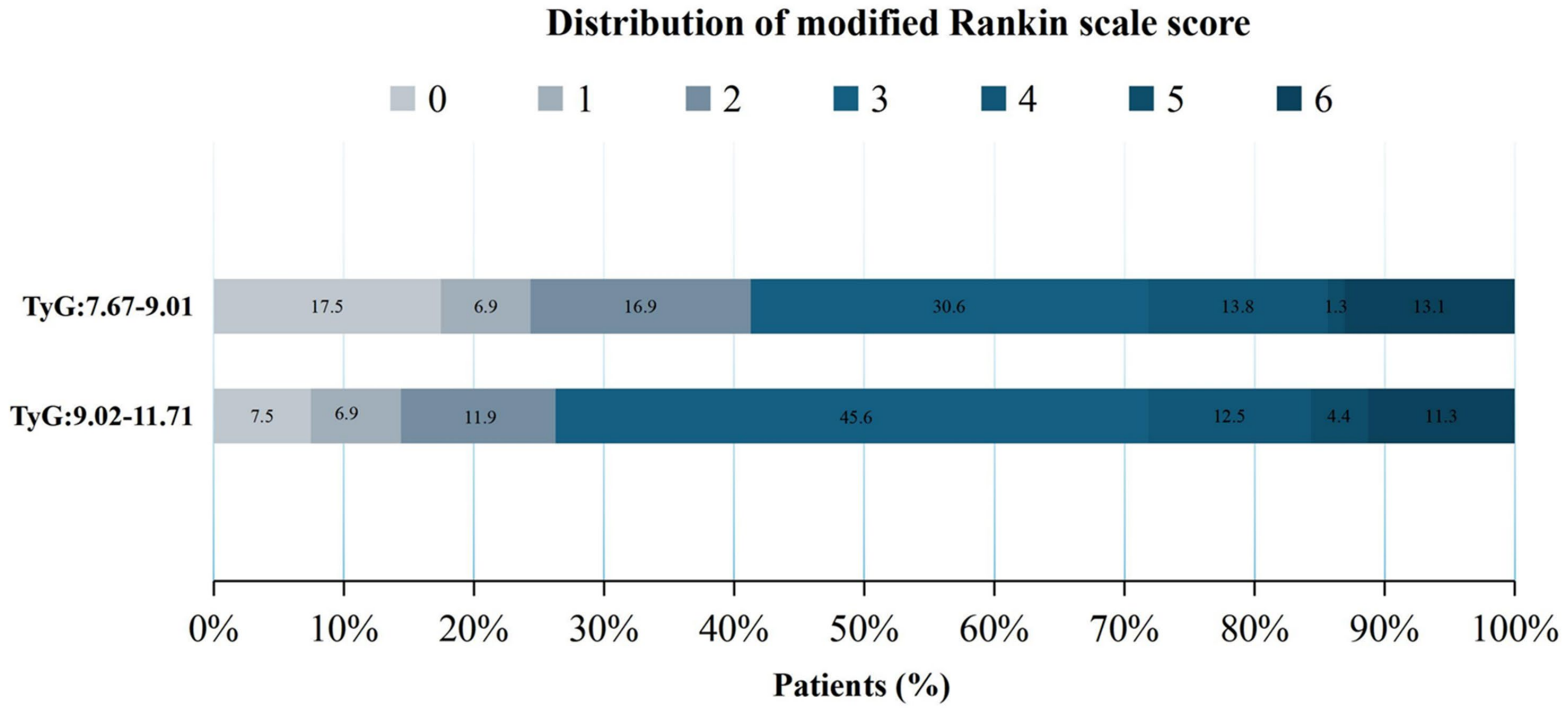
Distribution of the modified Rankin Scale (mRS) scores after 90 days across different groups.

The secondary clinical outcomes comprised the distribution of mRS scores at 90 days (Figure 2 illustrates the distribution of the mRS score in patients grouped by median TyG index), NIHSS scores at 24 h postoperatively, and NIHSS scores at the time of discharge.

No significant differences were observed between groups before covariate adjustment (*p* > 0.05) (Table 2). When analyzed with an adjusted model, a TyG index of 9.02–11.71 significantly increased the odds of a one-point improvement in mRS scores within 90 days after MT (Q2: aOR = 1.65, 95% CI, 1.06–2.59; *p* = 0.027). Furthermore, patients with higher TyG (Q2: 9.02–11.71) levels exhibited a significantly increased risk for higher NIHSS scores both at 24 h (Q2 vs. Q1: aOR = 2.13, 95% CI, 0.55–3.72; *p* = 0.009) and at the time of discharge (Q2 vs. Q1: aOR = 2.04, 95% CI, 0.11–3.96, *p* = 0.038), as indicated by the adjusted model. These significant results suggest that higher postoperative NIHSS scores in patients with higher TyG levels may indicate a poor prognosis.

### Safety outcomes

No statistically significant differences were observed between safety outcomes (including 90-day mortality, surgery-related complications, sICH within 24 h, and any intracranial hemorrhage within 48 h) and TyG levels in the unadjusted model (Table 2). Similarly, when analyzed with an adjusted model, no statistical significance was observed, indicating that the TyG index may not have a significant association with safety outcomes.

### Subgroup analyses

Subgroup analyses were conducted to elucidate the relationship between TyG and pivotal clinical endpoints across diverse demographic subgroups. A significant correlation was identified between TyG levels and functional independence at 90 days after thrombectomy, including the aged ≤62 years (OR = 0.540, 95% CI, 0.301–0.971), NIHSS score >13 (OR = 0.364, 95% CI, 0.180–0.735), hypertension (OR = 0.516, 95% CI, 0.286–0.931), internal carotid artery occlusion (OR = 0.489, 95% CI, 0.256–0.934), and large atherosclerosis (OR = 0.338, 95% CI, 0.174–0.656) groups, as detailed in Figure 1. These patients demonstrated stronger associations between elevated TyG levels and poorer functional outcomes. These findings may reflect increased metabolic and vascular vulnerability in these groups, warranting more focused monitoring and intervention.

## Discussion

This study revealed the effects of TyG levels on clinical outcomes in patients undergoing MT. A notable association was found between TyG levels and the likelihood of patients with acute large vessel occlusion in the anterior circulation achieving functional independence (mRS score ≤2) within 90 days after MT. Higher TyG levels were predictive of a decreased likelihood of functional independence, indicating their potential role as an adverse prognostic indicator. Although the NIHSS and ASPECT scores are the core indicators of stroke prognosis, the TyG index has the advantage of early accessibility (available on admission and independent of postoperative fluctuations in neurologic function) and reflection of systemic metabolic status (e.g., IR); thus, it can be used to supplement traditional neurologic function assessments. A notable strength of this study is its pioneering investigation of the correlation between TyG and the prognosis of patients with acute large vessel occlusion in the anterior circulation undergoing MT.

IR, defined by a diminished responsiveness of insulin-sensitive tissues to physiological insulin levels, is a key contributor to numerous conditions, such as ischemic stroke, type 2 diabetes mellitus, atherosclerosis, and metabolic syndrome (9–11). In particular, there is a robust correlation between IR and the incidence of ischemic stroke. A higher IR was linked to poorer clinical outcomes in patients with stroke undergoing thrombolytic therapy (aOR = 8.54, 95% CI, 1.67–43.35, *p* = 0.01) (22). Individuals with AIS and heightened IR may exhibit suboptimal responses to intravenous thrombolysis and could potentially benefit from more positive reperfusion strategies, such as MT. However, clinicians have yet to agree on a unified conclusion regarding the effects of IR on the efficacy of MT.

**Figure 3:**
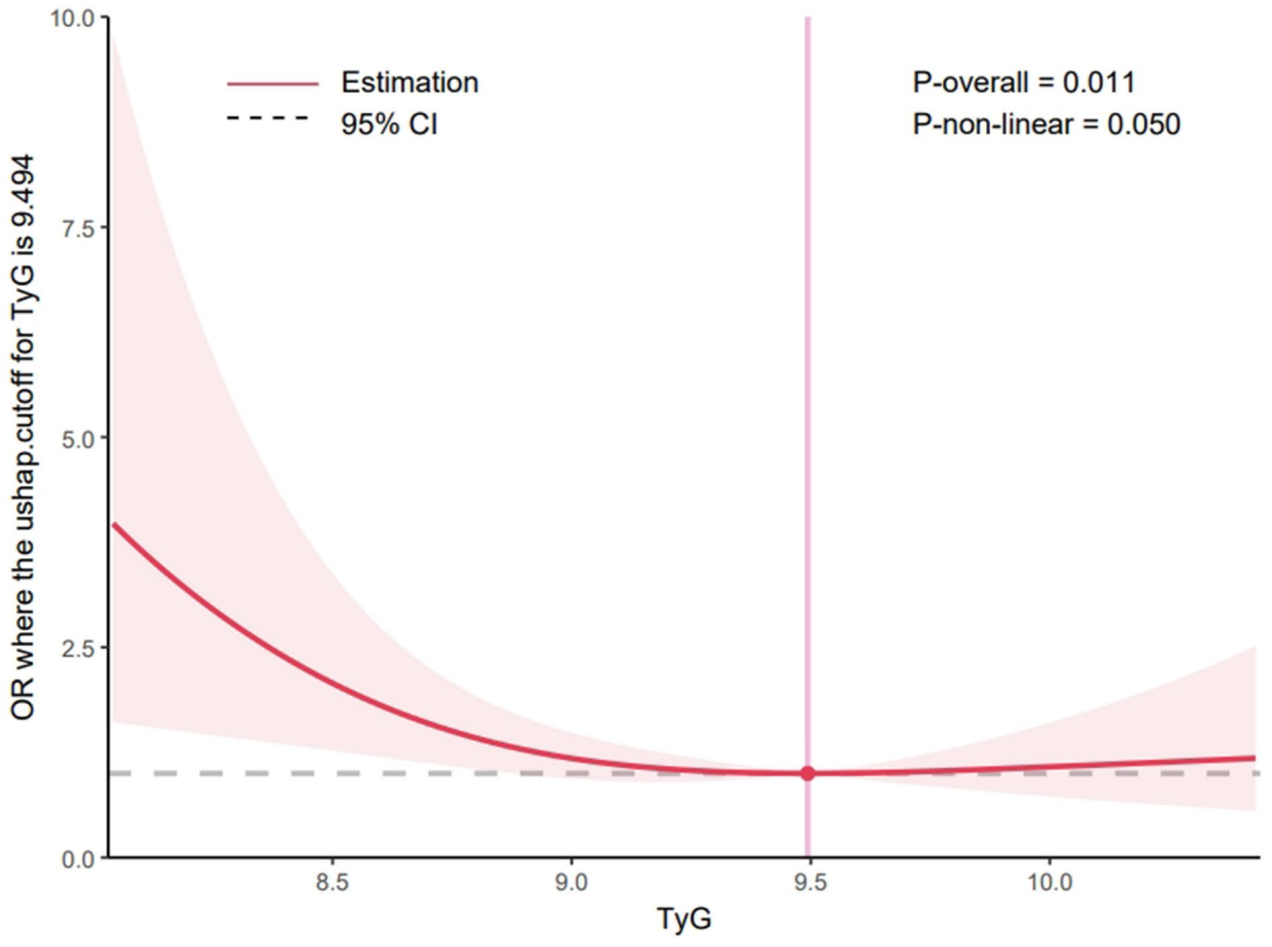
Restricted cubic spline curve for the TyG index odds ratio. Shaded ribbons denoting 95% confidence intervals. TyG index 9.494 was selected as the reference level represented by the vertical solid line. The horizontal dotted lines represent the odds ratio of 1.0.

A systematic review and meta-analysis revealed that patients with ischemic stroke with acute large vessel occlusion at admission and high glucose levels had an increased risk of sICH, worse neurological outcomes, and higher mortality following MT than normoglycemic patients (23). The TyG index has been investigated as a surrogate indicator for recognizing IR (14–17, 24). Therefore, this study used the TyG index as a novel approach to evaluate the prognosis of patients undergoing MT. This composite index, which integrates BG and TG levels, has a high sensitivity and specificity, suggesting its potential usefulness in identifying patients with IR (20). Additionally, TyG levels were positively related with a heightened likelihood of ischemic stroke; patients with higher TyG levels may have a higher incidence of ischemic stroke (OR = 1.37; 95% CI, 1.22–1.54, *p* < 0.05), and elevated TyG levels may significantly increase risks of stroke recurrence (OR = 1.50; 95% CI, 1.19–1.89, *p* < 0.05) and mortality (OR = 1.40; 95% CI, 1.14–1.71, *p* < 0.05) (25). A systematic review and meta-analysis further confirmed that the highest TyG group was independently linked to an increased risk of AIS (relative risk: 1.27; 95% CI, 1.24–1.29; I^2^ = 6%). A dose–response analysis further revealed a nonlinear trend in this association (26). In contrast, this study used restricted cubic spline regression modeling to examine the change in patients’ postoperative 90-day functional independence with TyG. When the TyG index was below 9.494, higher TyG levels were correlated with a reduced likelihood of achieving functional independence at 90 days. However, beyond this threshold, the proportion of patients achieving functional independence remained stable, despite further increases in TyG levels, as detailed in Figure 3. Among the secondary outcomes, patients with higher TyG levels had elevated NIHSS scores at 24 h postoperatively and discharge, indicating a worse neurological prognosis. These findings suggest a robust association between higher TyG levels and unfavorable prognosis post-MT. Recent research has also demonstrated a notable link between TyG and major adverse cardiovascular events in patients diagnosed with carotid atherosclerosis (27) and acute coronary syndrome (OR = 1.529, 95% CI, 1.001–2.061; *p* = 0.003) (19). These findings support TyG as a marker of acute adverse cardiovascular events. A study exploring the connection between TyG levels and poor prognosis after percutaneous coronary intervention in patients with acute STEMI found that elevated TyG levels were predictive of a heightened risk of major adverse cardiovascular and cerebrovascular events. In that study, patients were stratified into quartiles to identify predictors of these events (19). In contrast, the present study minimized the risk of difficult-to-interpret outcome indicators by dividing patients into two groups and adjusting for relevant covariates, thereby enhancing statistical significance. Focusing specifically on patients who underwent MT for acute anterior circulation occlusion, the findings indicate that higher TyG levels may be independently and significantly associated with unfavorable 3-month functional outcomes.

IR increases the risk of AIS, and the TyG index is an essential indicator for evaluating IR. Although the underlying pathological mechanisms linking TyG levels to ischemic stroke remain unclear, prevailing explanations suggest that vascular endothelial dysfunction, immune–inflammatory responses, and reduced aspirin responsiveness may contribute to this association (28–30). These mechanisms indicate that TyG levels may influence stroke onset and progression through multiple pathways. Therefore, additional investigations are required to deepen our understanding of the pathophysiological pathways linking TyG levels to ischemic stroke, and to explore potential applications of the TyG index in stroke prevention and treatment. Elevated TyG levels could inform decisions regarding thrombectomy candidacy, post-operative monitoring, and individualized care strategies. Future studies are needed to validate whether incorporating TyG into clinical practice improves patient outcomes.

### Study limitations

This study has certain limitations. Its retrospective, single-center design inherently introduced selective bias and the relatively small sample size limited statistical efficiency. Additionally, while we attempted to control for confounding factors, residual confounding (e.g., dietary habits, statin use) cannot be ruled out. The lack of external validation limits the generalizability of our findings, and future multi-center studies are required to confirm these results.

## Conclusion

This study demonstrates that TyG levels are independently and significantly correlated with functional outcomes in patients with acute anterior circulation stroke, 3 months after MT.

### What is already known on this topic

Patients with elevated TyG may have a higher incidence of ischemic stroke, and elevated TyG may significantly increase stroke recurrence and mortality. However, whether the TyG index can predict clinical outcomes in patients after acute anterior circulation MT remains unknown.

### What this study adds

Higher TyG levels may be associated with a worse 90-day functional independence prognosis, suggesting that TyG may serve as a negative predictor for evaluating the prognosis of patients. Higher postoperative NIHSS scores in patients with higher TyG may indicate a poor prognosis.

### How this study might affect research, practice or policy

The findings of this study present novel avenues for future research endeavors. Specifically, doctor should prioritize patients exhibiting elevated TyG indices, employing more aggressive therapeutic interventions and implementing rigorous monitoring protocols. Additionally, further investigation is warranted into the interplay between the TyG index and other biomarkers, as well as their combined influence on stroke prognosis, with the ultimate goal of enhancing patient outcomes.

## Data Availability

The original contributions presented in the study are included in the article/Supplementary material, further inquiries can be directed to the corresponding author.

## Glossary

TyG: triglyceride–glucose
TG: triglyceride
MT: mechanical thrombectomy
CVD: cardiovascular and cerebrovascular disease
mRS: modified Rankin Scale
aOR: adjusted odds ratios
IR: insulin resistance
CI: confidence intervals
AIS: acute ischemic stroke
sICH: symptomatic intracerebral hemorrhage
NIHSS: National Institutes of Health Stroke Scale
ASPECTS: Alberta Stroke Program Early Computed Tomography Score
ICH: intracranial hemorrhage
sICH: symptomatic intracerebral hemorrhage
IQR: interquartile range
BG: blood glucose
HOMA-IR: Homeostatic Model Assessment of Insulin Resistance
STEMI: ST-segment elevation myocardial infarction
CT: computed tomography
MRI: magnetic resonance imaging
TC: total cholesterol
HDL-C: high-density lipoprotein cholesterol
LDL-C: low-density lipoprotein cholesterol
SBP: systolic blood pressure
DBP: diastolic blood pressure

## References

[1] Li L Pan Y Wang M Jing J Meng X Jiang Y . Trends and predictors of myocardial infarction or vascular death after ischaemic stroke or TIA in China, 2007-2018: insights from China National Stroke Registries. Stroke Vasc Neurol. (2021) 6:214–21. doi: 10.1136/svn-2020-000503, PMID: 33127855 PMC8258052

[2] Barthels D Das H. Current advances in ischemic stroke research and therapies. Biochim Biophys Acta Mol basis Dis. (2020) 1866:165260. doi: 10.1016/j.bbadis.2018.09.012, PMID: 31699365 PMC6981280

[3] Goyal M Menon BK van Zwam WH Dippel DW Mitchell PJ Demchuk AM . Endovascular thrombectomy after large-vessel ischaemic stroke: a meta-analysis of individual patient data from five randomised trials. Lancet. (2016) 387:1723–31. doi: 10.1016/S0140-6736(16)00163-X, PMID: 26898852

[4] Jadhav AP Desai SM Jovin TG. Indications for mechanical thrombectomy for acute ischemic stroke: current guidelines and beyond. Neurology. (2021) 97:S126–36. doi: 10.1212/WNL.0000000000012801, PMID: 34785611

[5] Goyal N Tsivgoulis G Pandhi A Dillard K Katsanos AH Magoufis G . Admission hyperglycemia and outcomes in large vessel occlusion strokes treated with mechanical thrombectomy. J Neurointerv Surg. (2018) 10:112–7. doi: 10.1136/neurintsurg-2017-012993 28289148

[6] Osei E den Hertog HM Berkhemer OA Fransen PS Roos YB Beumer D . Increased admission and fasting glucose are associated with unfavorable short-term outcome after intra-arterial treatment of ischemic stroke in the MR CLEAN pretrial cohort. J Neurol Sci. (2016) 371:1–5. doi: 10.1016/j.jns.2016.10.003, PMID: 27871427

[7] Ginsberg HN Packard CJ Chapman MJ Borén J Aguilar-Salinas CA Averna M . Triglyceride-rich lipoproteins and their remnants: metabolic insights, role in atherosclerotic cardiovascular disease, and emerging therapeutic strategies-a consensus statement from the European atherosclerosis society. Eur Heart J. (2021) 42:4791–806. doi: 10.1093/eurheartj/ehab551, PMID: 34472586 PMC8670783

[8] Zhou X Kang C Hu Y Wang X. Study on insulin resistance and ischemic cerebrovascular disease: a bibliometric analysis via CiteSpace. Front Public Health. (2023) 11:1021378. doi: 10.3389/fpubh.2023.1021378, PMID: 36950100 PMC10025569

[9] Ding PF Zhang HS Wang J Gao YY Mao JN Hang CH . Insulin resistance in ischemic stroke: mechanisms and therapeutic approaches. Front Endocrinol (Lausanne). (2022) 13:1092431. doi: 10.3389/fendo.2022.1092431, PMID: 36589857 PMC9798125

[10] da Silva AA do Carmo JM Li X Wang Z Mouton AJ Hall JE. Role of hyperinsulinemia and insulin resistance in hypertension: metabolic syndrome revisited. Can J Cardiol. (2020) 36:671–82. doi: 10.1016/j.cjca.2020.02.066, PMID: 32389340 PMC7219403

[11] Di Pino A DeFronzo RA. Insulin resistance and atherosclerosis: implications for insulin-sensitizing agents. Endocr Rev. (2019) 40:1447–67. doi: 10.1210/er.2018-00141, PMID: 31050706 PMC7445419

[12] Hou XZ Lv YF Li YS Wu Q Lv QY Yang YT . Association between different insulin resistance surrogates and all-cause mortality in patients with coronary heart disease and hypertension: NHANES longitudinal cohort study. Cardiovasc Diabetol. (2024) 23:86. doi: 10.1186/s12933-024-02173-7, PMID: 38419039 PMC10903030

[13] Polovina M Krljanac G Ašanin M Seferović PM. Crouching tiger, hidden dragon: insulin resistance and the risk of atrial fibrillation. Eur J Prev Cardiol. (2020) 27:1931–3. doi: 10.1177/2047487320912626, PMID: 32237896

[14] Gastaldelli A. Measuring and estimating insulin resistance in clinical and research settings. Obesity (Silver Spring). (2022) 30:1549–63. doi: 10.1002/oby.23503, PMID: 35894085 PMC9542105

[15] Ramdas Nayak VK Satheesh P Shenoy MT Kalra S. Triglyceride glucose (TyG) index: a surrogate biomarker of insulin resistance. J Pak Med Assoc. (2022) 72:986–8. doi: 10.47391/JPMA.22-63, PMID: 35713073

[16] Khan S Qayyum S Khan MNA. Triglyceride-glucose index: a surrogate marker of homeostasis model assessment of insulin resistance to predict diabetic nephropathy. J Pak Med Assoc. (2024) 74:862–7. doi: 10.47391/JPMA.8505, PMID: 38783431

[17] Son DH Lee HS Lee YJ Lee JH Han JH. Comparison of triglyceride-glucose index and HOMA-IR for predicting prevalence and incidence of metabolic syndrome. Nutr Metab Cardiovasc Dis. (2022) 32:596–604. doi: 10.1016/j.numecd.2021.11.017, PMID: 35090800

[18] Nayak SS Kuriyakose D Polisetty LD Patil AA Ameen D Bonu R . Diagnostic and prognostic value of triglyceride glucose index: a comprehensive evaluation of meta-analysis. Cardiovasc Diabetol. (2024) 23:310. doi: 10.1186/s12933-024-02392-y, PMID: 39180024 PMC11344391

[19] Luo E Wang D Yan G Qiao Y Liu B Hou J . High triglyceride-glucose index is associated with poor prognosis in patients with acute ST-elevation myocardial infarction after percutaneous coronary intervention. Cardiovasc Diabetol. (2019) 18:150. doi: 10.1186/s12933-019-0957-3, PMID: 31722708 PMC6852896

[20] Guerrero-Romero F Simental-Mendía LE González-Ortiz M Martínez-Abundis E Ramos-Zavala MG Hernández-González SO . The product of triglycerides and glucose, a simple measure of insulin sensitivity. Comparison with the euglycemic-hyperinsulinemic clamp. J Clin Endocrinol Metab. (2010) 95:3347–51. doi: 10.1210/jc.2010-0288, PMID: 20484475

[21] Ajiboye N Yoo AJ. Biomarkers of technical success after embolectomy for acute stroke. Neurology. (2021) 97:S91–S104. doi: 10.1212/WNL.0000000000012800, PMID: 34785608

[22] Calleja AI García-Bermejo P Cortijo E Bustamante R Rojo Martínez E González Sarmiento E . Insulin resistance is associated with a poor response to intravenous thrombolysis in acute ischemic stroke. Diabetes Care. (2011) 34:2413–7. doi: 10.2337/dc11-1242, PMID: 21911778 PMC3198275

[23] Perez-Vega C Domingo RA Tripathi S Ramos-Fresnedo A Kashyap S Quinones-Hinojosa A . Influence of glucose levels on clinical outcome after mechanical thrombectomy for large-vessel occlusion: a systematic review and meta-analysis. J Neurointerv Surg. (2022) 14:neurintsurg-2021-017771. doi: 10.1136/neurintsurg-2021-017771, PMID: 34362794

[24] Gao W Wang J Chen Y Qiao H Qian X Xin Z . Discordance between the triglyceride glucose index and HOMA-IR in incident albuminuria: a cohort study from China. Lipids Health Dis. (2021) 20:176. doi: 10.1186/s12944-021-01602-w, PMID: 34865646 PMC8647334

[25] Yang Y Huang X Wang Y Leng L Xu J Feng L . The impact of triglyceride-glucose index on ischemic stroke: a systematic review and meta-analysis. Cardiovasc Diabetol. (2023) 22:2. doi: 10.1186/s12933-022-01732-0 36609319 PMC9825038

[26] Feng X Yao Y Wu L Cheng C Tang Q Xu S. Triglyceride-glucose index and the risk of stroke: a systematic review and dose-response meta-analysis. Horm Metab Res. (2022) 54:175–86. doi: 10.1055/a-1766-0202, PMID: 35276743

[27] Yu H Tao L Li YG Yang L Liu D Wang Y . Association between triglyceride-glucose index trajectories and carotid atherosclerosis progression. Cardiovasc Diabetol. (2023) 22:130. doi: 10.1186/s12933-023-01847-y, PMID: 37254140 PMC10230761

[28] Kaur R Kaur M Singh J. Endothelial dysfunction and platelet hyperactivity in type 2 diabetes mellitus: molecular insights and therapeutic strategies. Cardiovasc Diabetol. (2018) 17:121. doi: 10.1186/s12933-018-0763-3, PMID: 30170601 PMC6117983

[29] Wu H Ballantyne CM. Metabolic inflammation and insulin resistance in obesity. Circ Res. (2020) 126:1549–64. doi: 10.1161/CIRCRESAHA.119.315896, PMID: 32437299 PMC7250139

[30] Guo Y Zhao J Zhang Y Wu L Yu Z He D . Triglyceride glucose index influences platelet reactivity in acute ischemic stroke patients. BMC Neurol. (2021) 21:409. doi: 10.1186/s12883-021-02443-x, PMID: 34702218 PMC8549262

