# Correction: Prognostic relevance of the triglyceride–glucose index in patients after thrombectomy for acute anterior circulation occlusion

**DOI:** 10.3389/fneur.2026.1797694

**Published:** 2026-02-13

**Authors:** Zhongxiu Wang, Siyuan Wang, Chao Li, Kangjia Song, Xiaoyu Wu, Yu Jiang, Mingchen Zhang, Shouchun Wang

**Affiliations:** 1 Center for Rehabilitation Medicine, Department of Neurology, Zhejiang Provincial People's Hospital (Affiliated People's Hospital, Hangzhou Medical College), Hangzhou, Zhejiang, China; 2 Department of Neurology and Neuroscience, The First Hospital of Jilin University, Changchun, Jilin, China

**Keywords:** intravascular treatment, mechanical thrombectomy, ischemic stroke (acute), large vessel occlusion, triglyceride-glucose (TyG) index

In the published article, “Zhongxiu Wang” was erroneously assigned to affiliation ‘Department of Neurology and Neuroscience, The First Hospital of Jilin University, Changchun, Jilin, China’. The correct affiliation for author Zhongxiu Wang appears below:

“Center for Rehabilitation Medicine, Department of Neurology, Zhejiang Provincial People's Hospital (Affiliated People's Hospital, Hangzhou Medical College), Hangzhou, Zhejiang, China”

The original version of this article has been updated.

